# Development of a Repertoire and a Food Frequency Questionnaire for Estimating Dietary Fiber Intake Considering Prebiotics: Input from the FiberTAG Project

**DOI:** 10.3390/nu12092824

**Published:** 2020-09-15

**Authors:** Audrey M. Neyrinck, Julie-Anne Nazare, Julie Rodriguez, Romain Jottard, Sarah Dib, Monique Sothier, Laurie Van Den Berghe, Maud Alligier, Hélène Alexiou, Véronique Maquet, Sophie Vinoy, Stephan C. Bischoff, Jens Walter, Martine Laville, Nathalie M. Delzenne

**Affiliations:** 1 Metabolism and Nutrition Research Group, Louvain Drug Research Institute, UCLouvain, Université catholique de Louvain, 1200 Sint-Lambrechts-Woluwe, Belgium; audrey.neyrinck@uclouvain.be (A.M.N.); j.rodriguez@uclouvain.be (J.R.); romain.jottard@student.vinci.be (R.J.); sarah.dib@student.vinci.be (S.D.); 2 Centre de Recherche en Nutrition Humaine Rhône-Alpes, Université-Lyon, CarMeN Laboratory, Université Claude Bernard Lyon1, Hospices Civils de Lyon, 69310 Pierre Bénite, France; julie-anne.nazare@univ-lyon1.fr (J.-A.N.); monique.sothier@chu-lyon.fr (M.S.); ext-laurie.vandenberghe@chu-lyon.fr (L.V.D.B.); maud.alligier@chu-lyon.fr (M.A.); martine.laville@univ-lyon1.fr (M.L.); 3 Haute Ecole Léonard de Vinci, Institut Paul Lambin, 1200 Brussels, Belgium; helene.alexiou@vinci.be; 4 KitoZyme, 4040 Herstal, Belgium; v.maquet@kitozyme.com; 5 Mondelez Int. R&D, Nutrition Research, 91400 Saclay, France; sophie.vinoy@mdlz.com; 6 Institute of Nutritional Medicine, University of Hohenheim, 70593 Stuttgart, Germany; bischoff.stephan@uni-hohenheim.de; 7 Department of Agricultural, Food & Nutritional Science and Department of Biological Sciences, University of Alberta, Edmonton, AB T5J4P6, Canada; jwalter1@ualberta.ca; 8 APC Microbiome Ireland, School of Microbiology, and Department of Medicine, University College Cork, T12 YT20 Cork, Ireland

**Keywords:** FiberTAG study, food frequency questionnaire, prebiotic, inulin-type fructans, dietary fiber

## Abstract

Most official food composition tables and food questionnaires do not provide enough data to assess fermentable dietary fibers (DF) that can exert a health effect through their interaction with the gut microbiota. The aim of this study was to develop a database and a food frequency questionnaire (FFQ) allowing detailed DF intake estimation including prebiotic (oligo)saccharides. A repertoire of DF detailing total, soluble DF, insoluble DF and prebiotic (oligo)saccharides (inulin-type fructans, fructo-oligosaccharides and galacto-oligosaccharides) in food products consumed in Europe has been established. A 12 month FFQ was developed and submitted to 15 healthy volunteers from the FiberTAG study. Our data report a total DF intake of 38 g/day in the tested population. Fructan and fructo-oligosaccharides intake, linked notably to condiments (garlic and onions) ingestion, reached 5 and 2 g/day, respectively, galacto-oligosaccharides intake level being lower (1 g/day). We conclude that the FiberTAG repertoire and FFQ are major tools for the evaluation of the total amount of DF including prebiotics. Their use can be helpful in intervention or observational studies devoted to analyze microbiota–nutrient interactions in different pathological contexts, as well as to revisit DF intake recommendations as part of healthy lifestyles considering specific DF.

## 1. Introduction

Dietary fibers (DF) are non-digestible plant polysaccharides found in high amounts in fruits, vegetables and cereals. They have been shown to have important effects on human health, including preventing and alleviating constipation, reducing gastrointestinal cancer incidence and blood glucose levels, lowering blood cholesterol levels and blood pressure, and beneficially modulating gut microbiota [1]. The definition of DF, analytical methods allowing their characterization and quantification, as well as their effects on health and diseases have been extensively discussed from many years and were recently reviewed by Stephen et al. (2017) [2]. Importantly, the recommended amounts for consumption remain a matter of debate. Some criteria like DF solubility also merit to be revisited taking into account of their physiological effect. Indeed, although it is a widely accepted opinion that insoluble fibers are non-fermentable and soluble fibers are fermentable, almost all types of DF are fermentable, fully or to some extent [2]. The difference is that some DF are slowly fermented by the intestinal microbiota, whereas others are fermented more rapidly, and in some cases, to a limited extent. A classification according to the fermentability is difficult to establish since the evaluation has not been systematically performed for all DF. However, some general statements can be made: soluble-resistant (not viscous) oligosaccharides are highly fermentable in the colon. Among insoluble non-starch polysaccharides, the fermentability varies according to cereal from which it is extracted [2]. To obtain food labelling and dietary recommendations, analytical methods for DF have changed over the years since the definition has also evolved. The enzymatic-gravimetric official methods AOAC 985.29 and AOAC 991.43 are commonly used to determine total DF in food products as reported for example in the Belgian database Nubel, the Canadian Nutrient File and the Dutch Nevo database. The German Souci-Fachmann-Kraut table presents the results obtained only by enzymatic analyzes (AOAC methods or method of Southgate and Englyst) that mention the soluble and insoluble DF. Nubel (Belgium), Ciqual (France), McCance and Widdowson’s composition of foods (UK), Danish Food Composition Databank (Denmark) and Nevo (Netherlands) do not report soluble/insoluble DF content. All these conventional methods do not detect some fermentable DF such as low molecular weight fructo-oligosaccharides (FOS) or galacto-oligosaccharides (GOS) [3]. In addition, as illustrated by the French Ciqual table, data obtained from the scientific literature, from manufacturers or distributors without any reference, are taken into account in addition to the AOAC official methods. This illustrates that tables reporting quantitative data of all fiber types measured with adequate methodology are missing.

Improving human health through modulation of the microbiota is an evolving strategy that is part of a comprehensive approach to lifestyle wellness [4,5,6]. Gut microbiota-related health benefits are not yet included in the current dietary and public health recommendations but significant advances have been made to clarify and delineate the physiological effects of DF—linked to their fermentation—to take them into account for future recommendations [2,7,8]. Over 25 years ago, a class of compounds, termed prebiotics, were recognized for their ability to modulate host microbiota to the benefit of the host [9]. Fermentable DF that are prone to be used by the gut microbes are now clearly considered as prebiotics [4]. GOS and inulin-type fructans (ITF) that cover all β−2,1 linear fructans including native inulin (DP 2-60) and FOS (DP 2-8) fully fit with prebiotic definition. ITF and GOS currently dominate the prebiotic category as evidenced by numerous studies on their prebiotic effects, that was primarily linked to their capacity to increase *Lactobacillus* and/or *Bifidobacterium* spp. [4,10]. To date, numerous randomized controlled trials have shown health benefits of a variety of ITF and/or GOS in healthy individuals as well as in patient [5,10,11]. The health effects refer to mitigation of metabolic disorders (overweight and obesity, type 2 diabetes mellitus, metabolic syndrome and dyslipidemia, inflammation), promotion of gut health (in the context of constipation, bowel habit, diarrhea, irritable bowel syndrome, enterocolitis), modulation of immune function (in the context of allergy, infection, vaccination) and more recently improvement of brain functions and behavior (in the context of depression and anxiety) [4,12,13]. Those data support the interest to develop adequate tools to estimate ITF and GOS intake in human diet. Few papers relate ITF, GOS and fiber content in a subset of food products, measured upon official and adequate methodologies [14,15,16,17,18,19,20,21]. Therefore, we have built a dedicated repertoire detailing soluble, insoluble DF and prebiotic (oligo)saccharides (ITF and GOS) content in food products consumed in Europe. In addition, we have elaborated a related FFQ to propose its use for the evaluation of all types of DF.

## 2. Materials and Methods

### 2.1. Development of the FiberTAG Repertoire

The FiberTAG repertoire has been established firstly based on the Souci–Fachmann–Kraut German database and the Canadian Nutrient File. The interest of the German database is that this European database includes soluble and insoluble DF. Those databases were deeply completed by adding soluble versus insoluble DF content and for prebiotic (oligo)saccharide levels (ITF, FOS, GOS) in food products coming from 10 scientific publications [14,15,16,17,18,19,20,21,22,23].

### 2.2. Development of the FiberTAG FFQ

We developed a food frequency questionnaire (FFQ) that allows DF intake estimation including soluble DF, insoluble DF and prebiotic (oligo)saccharides. FiberTAG FFQ is a paper, self-administered questionnaire, following the rationale for building most FFQs (reviewed by Riordan et al. (2017) [24]). Consumption frequency referred to the last 12 month period.

### 2.3. Measurement of Dietary Fiber Intake in a Belgian Population

The FiberTAG study was chosen for assessment of DF intake via the new FiberTAG FFQ. The study is an interventional monocentric study established to characterize the fermentation of an insoluble fiber (chitin-glucan, Kiotransine^®^ from KitoZyme, Belgium versus placebo (maltodextrin from Cargill) by assessing the volatile compounds released in the breath. The primary outcome of the study (not reported in this paper) was the gut microbiota composition by Illumina sequencing of 16S rRNA. Sample size was estimated using the software PASS 14.0.7 and the paired mean power analysis test. Fifteen subjects were sufficient to detect with 94% of power a mean difference of 2% with an estimated standard deviation of difference of 2% (two sided) in the relative proportion of a specific gut bacterial genus. Sixteen subjects were included in the study to allow for a 10% drop out. Healthy subjects were recruited by the Center of Investigation in Clinical Nutrition (CICN), the platform from UCLouvain by displaying poster on the university site and by mails, by social networks, local newspaper and local flyers disposed in shops and doctor’s office from nearby cities. Subjects were pre-screened by a phone or mail questionnaire and if they apparently fit with the inclusion criteria listed in Supplementary Table S1. Fifteen subjects were selected respecting inclusion and exclusion criteria. All subjects gave their informed consent for inclusion before they participated in the study. The study was conducted in accordance with the Declaration of Helsinki, and the protocol was approved by the Ethics Committee (Comité d’Ethique Hospitalo-Facultaire UCLouvain/Cliniques Universitaires Saint-Luc Project; identification code: 2017/11SEP/436). Written informed consent was obtained from all subjects. The trial was carried out in accordance with the Good Clinical Practice (GCP) as required by the following regulations: the Belgian law of 7 may 2004 regarding experiments on the human persons and the EU Directive 2001/20/EC on Clinical Trials (registration at clinicaltrials.gov as NCT03494491; registered 11 April 2018).

Subjects’ height and weight were measured and their BMI was calculated. Subjects were asked to complete the FiberTAG FFQ. Detailed verbal and written instructions were provided by a dietician. An Excel tool was created from the FiberTAG repertoire and DF contents, including fructans, FOS, GOS, were calculated in all food products including composite foods and cooked meals (pastries, breads, etc.) by using food composition from classic recipes. The daily intake of different type of DF over the 12 month period was then calculated, taking into account frequency, portion size and seasonal consumption. Mean and median (with minimum and maximum) values of DF intake were calculated for each DF types and for each subject since there were sometimes up to 3 values for some types of DF depending on the sources mentioned in the new repertoire.

### 2.4. Data Integration and Statistical Analysis

Mean (±SD) were calculated for age, BMI and DF intakes; median (minimum and maximum) values were also used for fiber intakes. Pie charts, histogram and statistical analysis were conducted on GraphPad Prism 7.04.

## 3. Results

### 3.1. The FiberTAG Repertoire of Food Products Containing Dietary Fiber

Based on the German database, the Canadian database and the exhaustive literature, we obtained up to four different values for some food items and some categories of DF, depending on the methodology used for the DF analysis. Therefore, the concentration range (minimum and maximum values of DF content) was given and a median value was calculated (Supplementary Table S2). Latin names were detailed for vegetal items and preparation or cooking was detailed for some food products. The food products were classified into three main sources of DF (cereal products, vegetables and fruits) and each of them were subdivided; a fourth category was developed for chocolate products. In total, we obtained 15 subcategories (Supplementary Table S3). Among the 283 food items listed in total, the rates of missing values were 2% for TDF, 17% for SDF and IDF, 44% for ITF, 55% for FOS and 64% for GOS.

### 3.2. The FiberTAG Food Frequency Questionnaire (FFQ)

The screenshot of the FiberTAG FFQ is presented in Supplementary Figure S4. It presents a single frequency scale with typically six categories extending from ‘never’ to ‘more than once a day’. It consists into 3 DF-containing food groups (vegetables, fruits, cereal products), subdivided into 18 subcategories. FFQ considers ‘bread/pastries/cookies/cereal breakfast/rice/pasta’ for cereal products as well as ‘raw/fresh’, ‘cooked’, ‘juice’, ‘spreadable’ or ‘tinned’ for vegetables or fruits. A fourth section named “others” details condiments, chocolate, cooked meals, dairy products and beverages (vegetarian drinks and coffee substitutes) containing DF which account for DF in typical French and Belgian diets, since the questionnaires have been tested in those two countries as first intend. This results in a FFQ that includes 302 food items. The question of seasonal consumption has been addressed for some vegetables and fruits. The portion size was estimated using photographs from the published Belgian booklet (“Outil pour estimer la consommation alimentaire”, CIRIHA). An annex of this photobook was created for missing specific products following the same presentation and the same criteria than the original published booklet. Space for comments were let at the end of each food subcategories. The time required to complete this FFQ was on average one hour (determined on 10 volunteers in a preliminary study).

### 3.3. Daily DF Intakes

**Figure 1:**
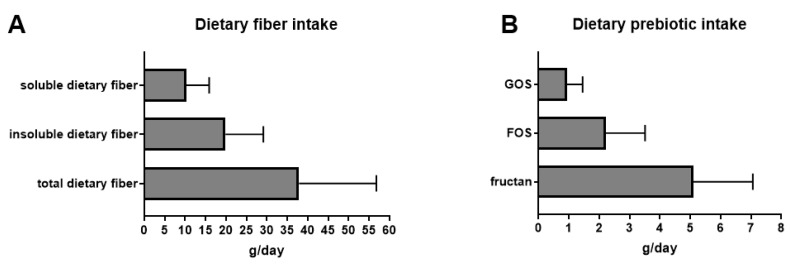
Mean daily intakes of dietary fiber (**A**) and prebiotic (**B**) calculated from the FiberTAG Food Frequency Questionnaire submitted to healthy volunteers using the FiberTAG repertoire. Values are means ± SD of the median values of daily fiber intake obtained for each volunteer and each fiber type. FOS, fructo-oligosaccharides; ITF, inulin-type fructans; GOS, galacto-oligosaccharides.

In total, 15 healthy subjects (7 men, 8 women) were recruited, with a mean age of 21 years and a mean BMI of 22 kg/m^2^. One of the volunteers was excluded because the answer to the questions were completely aberrant. Total DF intakes were calculated from the FiberTAG FFQ using the excel tools and the new FiberTAG repertoire (Supplementary Table S2). Figure 1 and Supplementary Table S5 show a high DF intake of 37.9 g/day (representing 1/3 of soluble and 2/3 of insoluble DF) over a 12 month period. ITF and FOS intakes were 5.1 and 2.2 g/day, respectively, whereas GOS intake was 0.9 g/day.

### 3.4. Principal Food Sources of Fiber Intakes

**Figure 2:**
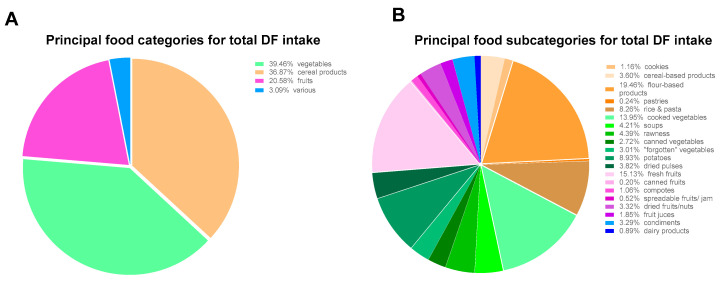
Contribution of food sources divided into categories (**A**) and subcategories (**B**) to the daily total dietary fiber intake. Data are determined from the FiberTAG Food Frequency Questionnaire submitted to healthy volunteers using the FiberTAG repertoire.

Figure 2A shows the distribution of the different types of DF grouped into 4 principal food categories while Figure 2B shows the food subcategories in the daily intake of total DF of subjects.

Vegetables was the largest contributor to total DF intake. Cereal products, and more specifically flour-based products, arrived in second position. Then, the fruit category, most often consumed as fresh fruits, contributed to 21% of total DF intake. Major food sources of naturally occurring ITF in Belgian diets were condiments (principally onions and garlic) which provided about 43%, whereas condiments and flour-based products provided each one third of daily FOS intake (Table 1). Cooked vegetables were the major source of GOS daily consumption.

## 4. Discussion

The novelty of this study was to propose a new repertoire and a new food questionnaire to better characterize the type and quality of DF intake. This evaluation of DF intake takes into account their fermentability and prebiotic properties involved in the management of cardiometabolic risk and health. Indeed, the FiberTAG repertoire is a new DF composition database detailing soluble versus insoluble DF, ITF, FOS, GOS content in addition to the total DF content of food products based on scientific recognized methods. An exhaustive literature search has been performed to deeply complete the database with prebiotic (oligo)saccharide levels (ITF, FOS, GOS) in food products using published scientific data. However, even though we found values of total DF for most of the food items, this repertoire highlighted several gaps for some categories of fiber (mostly FOS and GOS) and encouraged further additional analysis of prebiotic (oligo)saccharides. FFQ was chosen to assess DF intake in general population because it is inexpensive, simple to complete, and provide an overview of long-term food habits, widely used as the primary dietary assessment tool to epidemiologic studies [25]. By using the FiberTAG repertoire, data obtained from the FiberTAG FFQ indicated a total DF intake of 38 g/day in a small group of healthy young Belgian subjects. This value is higher than the estimated contribution for the average Belgian in the 2014–2015 Food Consumption Survey (18 g/day) and higher compared to previously reported intake for adults in Europe ranging from 16 to 24 g/day based on 24 h-recall or 3–7 days records. This estimation is also closer to the European (EFSA) and Belgian (CSS) recommendations, which ranges from a minimum of 25 g to ideally 30 g of fiber per day, in order to have significant health benefits [2]. This difference might be explained by a more adequate inclusion of fruits and vegetables intake in FFQ than with other methods, as previously reported [24]. To date, four studies have quantified dietary inulin and oligofructose intake: two studies were from the 1990s using food composition data from Van Loo et al. [14,21] whereas two studies were from the 2010s using a 23-item short FFQ developed to measure short-term inulin and oligofructose intake over the same 7 days [1,26]. ITF and FOS intakes were 5.1 and 2.2 g/day, respectively, whereas GOS intake was 0.9 g/day. Other features could affect the estimated fiber content such as the method of cooking or the presence of fruit or vegetable peel. The FiberTAG repertoire details such characteristics when the information is available. Overall, we can conclude that the influence of cooking or peeling are relatively limited confirming previous studies [14,18]. Even though it is determined in a limited sample size, this is the first study reporting GOS intake in a population. FOS intakes were already estimated in four studies [14,21,26,27]. American diets provided on average 2.5 g of oligofructose estimated from 24 h dietary recalls from 15,000 Americans and using only ITF contents given by Van Loo et al. (1995) [14]. It was reported in the recent study performed on 44 subjects in New Zealand that inulin or oligofructose intakes were around 3 g/day [27]. Here, intakes of FOS (2.2 g/day) were lower than in the US (2.5 g/day) [21]. ITF or FOS intakes estimated in the present study were at the lower end of the range of intakes across Europe (3.2–11.3 g/day) [14,26]. Our results are in accordance to those already reported for Belgium (ranging from 2.8 to 10.4 g/day) [14].

Our approach has the advantage and the drawback of all FFQ: The FiberTAG questionnaire requires an average of one hour to complete the form and it takes into account the eating habits over the year and the seasonality. A 24 h-recall or 3–7 day records could be more precise but also could lead to many biases, requires discipline and is time consuming. Its great advantage was the ability to characterize precisely the DF intake. We were thus able to highlight in this small population that when consumption of food is accounted for, vegetables are the most important sources of total DF, followed by cereal products (flour-based products). In line with previous studies reporting that wheat was the largest contributor to inulin and oligofructose intake [14,21,26], we have shown in the present study that cereal products are the principal contributors of FOS. When considering ITF intake, condiments, particularly onions and garlic (and in at a lesser extent chives and shallots), are the principal contributors of this prebiotic DF fibers assessed using the FiberTAG FFQ and the new FiberTAG database. This result may be explained by the high concentration of fructans in onions (average of 4.5 g/100 g) and garlic (16.7 g/100 g) versus their contents in cereal-based products (average of 1.6 g/100 g). The differences between studies may also relate to real differences in dietary intake between the populations linked to different dietary habits reported but also to differences in dietary assessment used, with the most comprehensive being the FiberTAG FFQ covering a large period (12 months) used here.

Our aim was to build a new tool for evaluation of prebiotic dietary fibers. Since the questionnaire has been submitted to a small cohort of healthy subjects, the interpretation of the data must be kept with caution. The use of a university population, with potentially higher levels of education and health consciousness, is therefore unrepresentative of the Belgian population. Despite this limitation, the (in)soluble DF intakes and prebiotic (oligo)saccharides intakes are intensively recorded and the first reported in Belgian population. The FiberTAG FFQ should be tested in other and larger cohorts and be adapted to specific dietary habits of populations following the aim of the studies.

The strengths of the present study include the development of the FiberTAG repertoire with a deep characterization about DF, in particular DF with prebiotic properties known to maintain health and to manage non-communicable diseases. This repertoire should be completed in the future by new analyses of prebiotic content in some food products. The FiberTAG FFQ was built using the gold standard described in the recent review aiming to identify methods to assess fruits and vegetables intake among children and adults in pan-European studies [24]. The present FFQ provides a tool with the possibility to associate specific fiber sources with outcomes since it focused on the main prebiotic (oligo)saccharides. A section of the FFQ details typically cooked French and Belgian meals, since the questionnaires have been tested in those two countries as first intended; this section is adaptable for DF intake estimation in another country. The FFQ, evaluated in the present study, is applicable for the Belgian adult population but might also be relevant for adult populations in other European countries with similar food patterns, in particular neighboring countries (Germany, France, Luxembourg, The Netherlands). Prebiotics are now used as functional ingredients in yoghurts and breakfast cereals. The formulation, use and availability of such food products vary between different countries [28]. Therefore, those wishing to use the FiberTAG FFQ should first identify this kind of fortified food products and record their intake to ensure accurate assessment of prebiotic (oligo)saccharides intakes [26].

## 5. Conclusions

In conclusion, the new FiberTAG repertoire and self-administered FiberTAG FFQ are both accurate tools suitable to assess DF intake including prebiotics in healthy adults. The food diary (7 day) may be the most accurate dietary method but they may change actual dietary intake and are inconvenient in many clinical trial settings. The 24 h recall requires qualified investigators and do not sufficiently reflect the real DF intake. FFQs may facilitate long-term dietary assessment, as long as they are rigorously validated. Therefore, despite the limitations discussed, we conclude that the FiberTAG FFQ developed here is a rapid and reliable method for measuring long-term (12 months) DF intake, including prebiotic (oligo)saccharide intakes. The high estimation of DF intake (38 g/day) might be explained by a more adequate inclusion of vegetables and fruits intake in FFQ than with other methods. The research of biomarkers—as proposed in the FiberTAG project [7]—could be used in the future to validate prebiotic DF intake, whereby the dependence of reference methods (food diary) for validation could be minimized. The FiberTAG project generates scientific knowledge that helps to take into account microbiota–nutrient interactions to establish DF intake recommendations as part of healthy lifestyles.

## Figures and Tables

**Table 1:** Principal food sources for daily prebiotic intake ^1^.

categories	ITF	FOS	GOS
cereal products	20.1%	30.8%	18.7%
vegetables	28.8%	29.7%	75.7%
fruits	8.7%	12.3%	0.0%
other	42.5%	27.1%	5.5%
**subcategories**			
cereal-based products	2.2%	0.2%	3.4%
cookies	3.2%	0.4%	0.0%
flour-based products	11.2%	30.1%	15.4%
pastries	0.0%	0.0%	0.0%
rice and pasta	3.4%	0.1%	0.0%
cooked vegetables	5.0%	8.0%	38.5%
soups	13.4%	16.1%	15.9%
rawness	0.5%	1.9%	0.3%
canned vegetables	1.8%	2.4%	0.1%
“forgotten” vegetables	5.82%	0.1%	0.0%
potatoes	1.0%	0.0%	0.0%
dried pulses	1.3%	1.3%	21.1%
fresh fruits	6.3%	8.5%	0.0%
canned fruits	0.0%	0.0%	0.0%
compotes	0.0%	0.0%	0.0%
spreadable fruits/jam	0.0%	0.0%	0.0%
dried fruits/nuts	0.0%	0.0%	0.0%
fruit juices	2.4%	3.8%	0.0%
condiments	42.5%	27.1%	5.6%
dairy products	0.0%	0.0%	0.0%

^1^ Contribution of food sources divided into categories and subcategories to the prebiotic intakes. Data are determined from the FiberTAG Food Frequency Questionnaire submitted to healthy volunteers using the FiberTAG repertoire. FOS, fructo-oligosaccharides; ITF, inulin-type fructans; GOS, galacto-oligosaccharides.

## Supplementary Materials

The following are available online at https://www.mdpi.com/2072-6643/12/9/2824/s1, Table S1, Inclusion and exclusion criteria, Table S2, the FiberTAG repertoire, Table S3, Categories and subcategories of the FiberTAG repertoire, Table S4, Daily intakes of dietary fiber, Figure S1, Screenshot of the FiberTAG Food Frequency Questionnaire.
