# Supplementary material for: Development of a Repertoire and a Food Frequency Questionnaire for Estimating Dietary Fiber Intake Considering Prebiotics: Input from the FiberTAG Project
Source: Nutrients. 2020 Sep 15;12(9):2824. doi: 10.3390/nu12092824 (PMC7551723; doi:10.3390/nu12092824)
Supplement: Supplementary file 1 [file nutrients-12-02824-s001.zip › supplementary-2/supplementary.pdf]

Supplementary Table S1. Inclusion and exclusion criteria

inclusion criteria	exclusion criteria
–Woman or man, aged of 18 to 40 years<br>–Body mass index (BMI) between 18 and 25 kg/m^2<br>–In good general health as evidenced by medical history and physical examination<br>–Non-smoker<br>–Caucasian<br>–For women: use of highly effective contraception<br>–H_2 – producer as evidenced by the screening test<br>–Provision of signed and dated informed consent form<br>–Stated willingness to comply with all study procedures and availability for the duration of the study	–Subjects presenting gastro-intestinal disorders such as ulcers, diverticulitis and inflammatory bowel diseases<br>–Subject presenting allergy or food intolerance (lactose, gluten,…)<br>–Subjects with psychiatric problems and/or using antipsychotics<br>–Current or recent (< 4 weeks) intake of antibiotics, probiotics, prebiotics, fiber complement, and/or any products modulation gut transit<br>–Feeding particular diet such as vegetarian diet or hyper protein diet<br>–Chronic intake of drug, excepted contraceptive drug<br>–Pregnant or lactating woman or woman who did not use highly effective contraception<br>–Subjects who drink more than 3 glasses of alcohol per day (> 30 g of alcohol per day)<br>–Subjects having participated to another clinical trial 1 month before the screening test visit<br>–Subjects presenting an allergy or intolerance to one component of the product tested

Supplementary Table S3. Categories and subcategories of the FiberTAG repertoire

Vegetables	Fruits	Cereal products	Chocolate products
Legumes	Fresh fruits	Cookies	Chocolate
Roots and tubers	Dried fruits	Breads	
Other vegetables	Nuts	Crude cereals	
	Seeds	Processed cereals	
		Flours	
		Rice	
		Pasta	

Supplementary Table S4. Daily intakes of dietary fiber[1]

Dietary fiber		Daily intakes (g)
**total**	*Minimum*	10.8
	Maximum	76.4
	**Median**	37.2
**insoluble**	*Minimum*	6.4
	Maximum	41.7
	**Median**	18.1
**soluble**	*Minimum*	3.4
	Maximum	24.3
	**Median**	9.1
**ITF**	*Minimum*	2.6
	Maximum	9.4
	**Median**	4.7
**FOS**	*Minimum*	1
	Maximum	5.5
	**Median**	1.7
**GOS**	*Minimum*	0.2
	Maximum	1.7
	**Median**	0.9

[1] Minimum, maximum and median values of daily fiber intakes calculated from the FiberTAG Food Frequency Questionnaire submitted to healthy volunteers using the FiberTAG repertoire. FOS, fructo-oligosaccharides; ITF, inulin-type fructans; GOS, galacto-oligosaccharides

Supplementary Figure S1. Screenshot of the FiberTAG Food Frequency Questionnaire

FiberTAG

Vegetables

Cooked vegetables –soups - rawness – canned vegetables –old vegetables –potatoes –dry vegetables

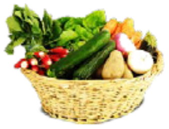

Cooked vegetables	Never or < 1 time a month If yes, how much ?	1-2 times a month	1-2 times a week	3-5 times a week	1 time a day	> 1 time a day If yes, how much ?	Picture	Indicate a letter (A-B-C-D-E-F-G-H-I)	Seasonal consumption ?If yes, specify the season	In what form ?
Asparagus		☐	☐	☐	☐		A207			
Eggplant		☐	☐	☐	☐		M83			Whith or whithout peel ?
Zucchini		☐	☐	☐	☐		M83			Whith or whithout peel ?
Pepper		☐	☐	☐	☐		M83			Whith or whithout peel?
Brussels sprouts		☐	☐	☐	☐		M75			
Chinese leaves(Pak-Choi)		☐	☐	☐	☐		A208			
White cabbage		☐	☐	☐	☐		A208			
Green cabbage		☐	☐	☐	☐		A208			
Curly cabbage		☐	☐	☐	☐		A208			
Cauliflower		☐	☐	☐	☐		M76			
